# Anticipatory approach for dynamic and stochastic shipment matching in hinterland synchromodal transportation

**DOI:** 10.1007/s10696-021-09428-5

**Published:** 2021-08-06

**Authors:** Wenjing Guo, Bilge Atasoy, Wouter Beelaerts van Blokland, Rudy R. Negenborn

**Affiliations:** 1 Department of Maritime and Transport Technology, Delft University of Technology, Delft, The Netherlands; 2 Department of Analytics, Operations and Information Technologies, University of Quebec at Montreal and CIRRELT, Montreal, Canada

**Keywords:** Synchromodal transportation, Dynamic shipment matching, Stochastic spot requests, Anticipatory approach

## Abstract

This paper investigates a dynamic and stochastic shipment matching problem faced by network operators in hinterland synchromodal transportation. We consider a platform that receives contractual and spot shipment requests from shippers, and receives multimodal services from carriers. The platform aims to provide optimal matches between shipment requests and multimodal services within a finite horizon under spot request uncertainty. Due to the capacity limitation of multimodal services, the matching decisions made for current requests will affect the ability to make good matches for future requests. To solve the problem, this paper proposes an anticipatory approach which consists of a rolling horizon framework that handles dynamic events, a sample average approximation method that addresses uncertainties, and a progressive hedging algorithm that generates solutions at each decision epoch. Compared with the greedy approach which is commonly used in practice, the anticipatory approach has total cost savings up to 8.18% under realistic instances. The experimental results highlight the benefits of incorporating stochastic information in dynamic decision making processes of the synchromodal matching system.

## Introduction

Hinterland transportation is the movement of shipments between deep-sea ports and inland terminals by trucks, trains, barges, or any combination of them (SteadieSeifi et al. 2014). Typically, a hinterland transport system is made up of multiple stakeholders that interact with each other, including network operators, shippers, carriers, terminal operators, and institutional authorities (Crainic et al. 2018). Network operators (e.g., logistics service providers and alliances formed by multiple carriers) control the transport system. Shippers (e.g., manufacturers, ocean carriers, and freight forwarders) generate freight transport demand and outsource transport activities to network operators. Carriers (e.g., truck, train, and barge companies) provide transport services and supply timely transport capacity to network operators. Terminal operators handle transshipment operations at terminals. Institutional authorities (e.g., governments and public administrations) charge tax, give incentives, and regulate transport activities to network operators, such as the charging of carbon emissions.

As shippers become more time-sensitive that require shipments to be delivered within tight time windows, trucks are used more often which contributes to road traffic congestion, transport costs, and carbon emissions (Demir et al. 2016). However, due to the increasing environmental issues and the enforced regulations, companies in the transport industry are required to control carbon emissions (Demir et al. 2016). Synchromodal transportation, as an emerging and attractive concept, aims to manage different types of shipments considering the trade-off among costs, delays, and emissions through integrated real-time planning and synchronization of activities (Giusti et al. 2019). Under synchromodality, shippers only specify shipments’ origin, destination, volume, release time, and due time, and leave the choice of modes, routes, and departure and arrival times to network operators. For example, for time-sensitive shipments, network operators can assign trucks for transportation; but if time available, barges, trains or barge-truck can be assigned taking into account their impact on costs, time, and emissions.

With the development of digitization in the logistics industry, increasing online booking platforms have appeared in freight transportation, such as Uber Freight, Quicargo, and Maersk Spot. In this paper, we consider a synchromodal matching platform owned by a network operator (e.g., European Gateway Services or Contargo) that receives contractual and spot shipment requests from shippers and receives time-scheduled services (e.g., trains) and departure time-flexible services (e.g., trucks) from carriers. The platform aims to provide optimal matches between shipment requests and transport services over a given planning horizon. Having a match between a shipment and a service means that the shipment will be transported by the service from the service’s origin terminal to the service’s destination terminal. The platform combines the matched services into shipments’ itineraries.

In practice, container transport companies receive shipment requests from both long-term contracts and spot markets (Meng et al. 2019). Different from the contractual requests received from large shippers whose information is known before the operational planning horizon, the information of spot requests is unknown and revealed dynamically (Guo et al. 2020). The demand from the spot market is influenced by many factors, such as global economy, seasonality, fluctuations of freight rate, and competitions from other companies (Wang and Meng 2021). Due to the capacity limitation of multimodal services, the capacity assigned to current requests will be unavailable for future requests which might be more profitable. Thanks to the advancements in information technologies, such as increased use of sensors in transport infrastructures, communication technologies, open data sources, and data analytics, exploiting stochastic information of spot requests is increasingly achievable (Gendreau et al. 2016). With the stochastic information, network operators might hold some barge and train capacities available for spot requests which are predicted to be more profitable.

In this paper, we define the matching of shipments and services under spot request uncertainty with the aim to minimize total costs over a given planning horizon as the dynamic and stochastic shipment matching (DSSM) problem. The complexity of the DSSM problem lies in three aspects. First, spot requests arrive in the platform in real-time which calls for a dynamic approach that handles dynamic events. Second, the stochastic information of spot requests is available which calls for a stochastic approach that addresses uncertainties. Third, the computation complexity of the optimization problem calls for an efficient algorithm that generates timely solutions at each decision epoch.

In the literature, Guo et al. (2020) developed a myopic approach to solve the DSSM problem which does not consider the stochasticity of spot requests. The myopic approach involves a rolling horizon framework that handles dynamic events and a preprocessing-based heuristic algorithm that generates timely solutions at each decision epoch. As an extension of Guo et al. (2020), this paper proposes an anticipatory approach to incorporate the stochastic information of spot requests in the dynamic shipment matching processes. The anticipatory approach involves a sample average approximation method that addresses spot request uncertainties and a progressive hedging algorithm that solves the deterministic formulations at each decision epoch of a rolling horizon framework.

The remainder of this paper is structured as follows. We briefly review the relevant literature and specify our contributions in Sect. 2. In Sect.  3, we describe the DSSM problem. In Sect. 4, we design the rolling horizon framework, the sample average approximation method, and the progressive hedging algorithm. In Sect. 5, we describe the experimental setup, and present the experimental results. Finally, in Sect. 6, we provide concluding remarks and directions for future research.

## Literature review

In the past decades, because of economic factors and environmental concerns, different management concepts have appeared in the literature and in the logistics industry: multimodal, intermodal, co-modal and synchromodal transportation. While multimodality refers to the utilization of multiple modes, intermodality emphasizes the utilization of standardized loading units (i.e., containers), namely the vertical integration of different modes (SteadieSeifi et al. 2014); co-modality focuses on the optimal and sustainable utilization of different modes on their own or in combination, namely the horizontal integration of different modes. As an extension of intermodality and co-modality, synchromodality adds the (real-time) flexibility in planning when disturbances happen (Giusti et al. 2019).

The implementation of synchromodal transportation relies on collaboration among stakeholders, information technologies, and integrated planning at different decision levels. Typically, synchromodal transport planning can be divided into three levels: strategic, tactical, and operational level. While strategic and tactical planning focus on physical network design (e.g., hub location) and service network design (e.g., service selection, service frequency) in long and medium time horizons, operational planning deals with the routing of shipments under dynamic and stochastic environments (Giusti et al. 2019).

In the literature, the majority of the studies (e.g., Ayar and Yaman 2011; Chang 2008; Moccia et al. 2010; van Riessen et al. 2014) related to synchromodal transport planning are conducted in a static and deterministic environment, namely, all the inputs are known beforehand and decisions do not change once they are set. However, in practice, there are many sources of uncertainties in synchromodal transportation, such as demand uncertainty. With the growing amount of historical data, the stochastic information about uncertainties is available. Incorporating stochastic information in decision-making processes has been proven to have better performance than the corresponding myopic approaches in many fields, such as vehicle routing problems (Albareda-Sambola et al. 2014) and dial-a-ride problems (Schilde et al. 2011).

In the field of stochastic synchromodal transport planning, Demir et al. (2016) studied a green intermodal service network design problem with demand and travel time uncertainties. In this study, the origins, destinations, time windows of shipments are known in advance, but the actual demand (i.e, the number of containers) is uncertain. A sample average approximation method was proposed to generate robust plans. Hrušovský et al. (2016) proposed a hybrid approach combining a deterministic model with a simulation model to investigate an intermodal transport planning problem with travel time uncertainty. Sun et al. (2018) established a fuzzy chance-constrained mixed integer nonlinear programming model to describe rail service capacity uncertainty and road traffic congestion. Generally, stochastic transport planning problems have the probability distributions of random variables and the optimization process is performed before their realization. The transport plan will not be updated after the realization, thus, it is often referred to as a-priori optimization (Ritzinger et al. 2015).

The trend towards digitalization in transportation allows gathering real-time information and thus dynamic decision making. In synchromodal transportation, some input data are revealed during the execution of the plan. The most common dynamic events are the arrival of new shipment requests, but demands and travel times are possible dynamics as well. In the literature, Li et al. (2015) presented a receding horizon intermodal container flow control approach to deal with the dynamic transport demands and dynamic traffic conditions. Mes and Iacob (2015) considered the real-time planning of shipment requests under a synchromodal network with the objective to minimize costs, delays, and emissions. van Heeswijk et al. (2016) proposed an online planning algorithm to schedule the transport of less than truckload freight via intermodal networks. Guo et al. (2020) developed a rolling horizon approach to handle shipment requests that arrive dynamically in a synchromodal matching platform.

The advances in information and communication technologies as well as the computing power allow the incorporation of stochastic information of future events in dynamic decision-making processes. Approaches for dynamic and stochastic transport planning problems can be divided into two categories: methods based on preprocessed decisions and methods based on online decisions. Solution approaches in the first group (preprocessed decisions) determine the values and policies of decision making before the execution of the transport plan (Ritzinger et al. 2015). Therefore, possible states need to be constructed in advance and evaluated based on possible dynamic events and stochastic information over a planning horizon. For example, van Riessen et al. (2016) designed a decision tree to derive real-time decision rules for suitable allocation of shipment requests to services. Rivera and Mes (2017) proposed an algorithm based on approximate dynamic programming to tackle the curse of dimensionality of a Markov decision process model. The second group (online decisions) focuses on the computation when a dynamic event occurs. Specifically, decisions are made online with respect to the current system state and the available stochastic information. SteadieSeifi (2017) proposed a rolling horizon approach to handle dynamic demands. At each iteration of the rolling horizon framework, the author proposed a scenario-based two-stage stochastic programming model to incorporate the stochastic information of future demands.

In this paper, we investigate the dynamic and stochastic shipment matching (DSSM) problem in synchromodal transportation at the operational level. The formulation characteristics of the DSSM problem include: (1) contractual and spot shipment requests; (2) stochastic information of spot requests; (3) unsplittable shipments, i.e., a shipment should be delivered as a whole; (4) soft time windows, i.e., delay in delivery is available but with a penalty; (5) capacitated and time-scheduled barge and train services; (6) departure time-flexible truck services with time-dependent travel times; (7) transshipment operations at terminals; (8) minimizing generalized costs which consist of transport costs, delay costs, and carbon tax over a planning horizon. The formulation characteristics, solution approaches, and experimental size of related articles are summarized in Table 1.

**Table 1:** Formulation characteristics, solution approaches and experiment size of related articles

Articles	Dynamic information$^{{\mathrm{a}}}$	Stochastic information$^{{\mathrm{a}}}$	Integrity	Time windows	Barge/train services $^{{\mathrm{b}}}$	Truck services	Transshipment	Objectives$^{{\mathrm{c}}}$	Methods$^{{\mathrm{d}}}$	Maximum instance size$^{{\mathrm{e}}}$
Demir et al. (2016)	–	Demand, travel times	Splittable	Soft	Capacitated	Flexible	$\checkmark$	C, D, E	SAA	I-20-100-20; I-20-250-5; I-20-500-1
Hrušovský et al. (2016)	–	Travel times	Splittable	Soft	Capacitated	Flexible	$\checkmark$	C, D, E	SO	I-20-250-20
Sun et al. (2018)	–	Service capacity	Unsplittable	Soft	Capacitated	Flexible, time-dependent	$\checkmark$	C, D, E	MILP	I-12-25-10
Li et al. (2015)	Demand, travel times	–	Splittable	–	Capacitated	Flexible	$\checkmark$	C	RHA	I-6-54-1
Mes and Iacob (2015)	Shipment requests	–	Unsplittable	Soft	Capacitated	Flexible	$\checkmark$	C, D, E	GA	I-6-110-1728
van Heeswijk et al. (2016)	Shipment requests	-	Unsplittable	Hard	Uncapacitated	Flexible	$\checkmark$	C, D, E	CA	I-37-110-1006
Guo et al. (2020)	Shipment requests	–	Unsplittable	Soft	Capacitated	Flexible, time-dependent	$\checkmark$	C, D, E	RHA	I-10-116-1600
van Riessen et al. (2016)	Shipment requests	Demand	Splittable	Soft	Capacitated	Flexible	–	C, D	DT	I-2-4-20
Rivera and Mes (2017)	Shipment requests	Shipment requests	Splittable	Hard	Capacitated	Flexible	–	C	ADP	I-12-29-40
SteadieSeifi (2017)	Demand	Demand	Splittable	Hard	Capacitated	Scheduled	–	C	RHA, STSP	I-20-400-200
This paper	Shipment requests	Shipment requests	Unsplittable	Soft	Capacitated	Flexible, time-dependent	$\checkmark$	C, D, E	RHA, SAA, PHA	I-10-116-1600

$^{{\mathrm{a}}}$ Information of shipment requests consists of shipments’ origin, destination, container volume (i.e., demand), announce time, release time, and due time

$^{{\mathrm{b}}}$ All the articles consider time-scheduled barge or train services

$^{{\mathrm{c}}}$ *C* Costs, *D* Delays, *E* Emissions

$^{{\mathrm{d}}}$ *SAA* Sample average approximation method, *SO* Simulation-optimization, *HA* Hybrid algorithm, *MILP* Mixed integer linear programming, *RHA* Rolling horizon approach, *GA* Greedy approach, *CA* Consolidation algorithm, *DT* Decision trees, *ADP* Approximate dynamic programming, *STSP* Scenario-based two-stage stochastic programming, *PHA* Progressive hedging algorithm

$^{{\mathrm{e}}}$ Instances follow naming convention of I-a-b-c where a represents the number of terminals, b is the number of services, and c is the number of shipment requests

Our work has three main contributions to the literature. First, we introduce the stochasticity of spot requests in the dynamic shipment matching processes. Second, we propose an anticipatory approach to solve the problem under realistic instances in a reasonable time. The anticipatory approach uses a sample average approximation method to address spot request uncertainty and applies a progressive hedging algorithm to get solutions at each decision epoch of a rolling horizon framework. This approach enables to consider a large set of scenarios (within 1 min of computation time) to more accurately represent the stochasticity and this in turn increases the benefits of incorporating stochastic information in dynamic decision-making processes. Third, thanks to the above developed methodologies we propose a platform in which companies can manage different types of shipments (e.g., time-sensitive shipments) under a synchromodal network considering the trade-off among costs, delays, and emissions. Such a platform provides the means for a more efficient, effective and sustainable decision-making framework for transportation systems.

## Problem description and preprocessing procedures

In this section, we first describe the DSSM problem in detail, and then briefly present the preprocessing procedures designed to reduce the computational complexity.

### Problem description

We consider an online matching platform that receives contractual and spot shipment requests from shippers, receives time-scheduled and departure-time flexible services from carriers, and receives unlimited handling services (i.e., loading and unloading) from terminal operators. Let *N* be the set of terminals. Let $lc^{\mathrm {barge}}_i$, $lc^{\mathrm {train}}_i$, $lc^{\mathrm {truck}}_i$ be the loading/unloading cost coefficient of barge, train, and truck services at terminal $i\in N$, respectively. Let $lt^{\mathrm {barge}}_i$, $lt^{\mathrm {train}}_i$, $lt^{\mathrm {truck}}_i$ be the loading/unloading time of barge, train, and truck services at terminal $i\in N$, respectively. Let $c^{\mathrm {storage}}_i$ be the storage cost coefficient at terminal $i\in N$. The $CO_2$ emissions-related cost coefficient is set as $c^{\mathrm {emission}}$.

Let *R* be the set of shipment requests. Each shipment request $r \in R$ is characterized by its announce time $t_r^{\mathrm {announce}}$ (i.e., the time when the platform receives the request), release time $t_{r}^{\mathrm {release}}$ (i.e., the time when the shipment is available for hinterland transportation) at origin terminal $o_{r}$, due time $t_{r}^{\mathrm {due}}$ (i.e., the time that the shipment needs to be delivered) at destination terminal $d_{r}$, expiry date $t_r^{\mathrm {expire}}$ (i.e., the time that the matching decisions for request *r* cannot be further postponed), and container volume $u_{r}$. Delay in delivery is available but with a penalty cost per container per hour overdue $c_r^{\mathrm {delay}}$.

Requests *R* can be divided into two groups: contractual requests $R^{\mathrm {contract}}$ and spot requests $R^{\mathrm {spot}}$. While $R^{\mathrm {contract}}$ are known beforehand, $R^{\mathrm {spot}}$ are unknown and revealed dynamically. However, the probability distributions $\{\pi _o, \pi _d, \pi _u, \pi _{t^{\mathrm {announce}}}, \pi _{t^{\mathrm {release}}}, \pi _{t^{\mathrm {due}}}, \pi _{t^{\mathrm {expire}}}\}$ of spot requests’ origin, destination, volume, announce time, release time, due time, and expiry date are assumed available from historic data. In addition, shippers require their shipments to be transported as a whole, and ask to receive the transport plan before shipments’ release time, namely the expiry date is equal to the release time, $t^{\mathrm {release}}_r=t^{\mathrm {expire}}_r$.

Let *V* be the set of transport services, all the services are received before the planning horizon. According to the time schedules, services can be divided into two groups:

- *Time-scheduled barge and train services*. Each barge or train service $v \in V^{\mathrm {barge}} \cup V^{\mathrm {train}}$ is characterized by its departure time $t^{\mathrm {depature}}_v$ at origin terminal $o_v$, arrival time $t^{\mathrm {arrival}}_v$ at destination terminal $d_v$, free capacity $U_v$, transport cost $c_v$ and carbon emissions $e_v$.
- *Departure time-flexible truck services*. We view each truck service as a fleet of trucks which has flexible departure times and an unlimited capacity. Thus, a truck service might have multiple departure times for different shipments. Due to traffic congestion at several time periods throughout a day, the travel time of truck services is time-dependent (Ichoua et al. 2003). Therefore, each truck service $v \in V^{\mathrm {truck}}$ is characterized by its origin terminal $o_v$, destination terminal $d_v$, time-dependent travel time function $t_v^{\mathrm {truck}}(\tau )$, transport cost $c_v$, and carbon emissions $e_v$.

The objective of the platform is to provide optimal online matches in total costs between shipment requests and transport services over a planning horizon *T*. The total costs consist of transit costs generated by using services, transfer costs and storage costs generated at transshipment terminals, penalty costs caused by delay in delivery, and carbon tax charged for services’ carbon emissions.

### Preprocessing procedures

In this section, we briefly present the preprocessing procedures that aim to reduce the computational complexity of the DSSM problem by identifying infeasible matches between shipments and services. It consists of two steps: the preprocessing of feasible path and the preprocessing of feasible matches.

- *Preprocessing of feasible path*. We define a path *p* as a combination of services in sequence. A path *p* is feasible if the services inside a combination satisfy time-spatial compatibility. Specifically, for two consecutive services $v_i,v_{i+1}$ within path *p*, the destination of service $v_i$ must be the same as the origin of service $v_{i+1}$; the arrival time of service $v_i$ must be earlier than the departure time of service $v_{i+1}$ minus loading and unloading time at transshipment terminal $d_{v_i}$. The set *P* denotes the collection of feasible paths.
- *Preprocessing of feasible matches*. A match (*r*, *p*) means shipment *r* will be transported by path *p* from its origin to its destination. A match between request $r \in R$ and path $p=\left[ v_1,...,v_l\right] \in P$ is feasible if it satisfies time-spatial compatibility:
  - *Spatial compatibility*. The origin terminal of shipment request *r* should be the same as the origin of service $v_1$; the destination of request *r* should be the same as the destination of service $v_l$.
  - *Time compatibility*. The release time of request *r* should be earlier than the departure time of service $v_1$ minus loading time at origin terminal $o_r$.
  Let $P_r$ be the set of feasible paths for request *r*, and let $c_{rp}$ denote the costs of matching request *r* with path *p* including transport costs, delay costs and carbon tax. The details of the preprocessing procedures are presented in Guo et al. (2020).

**Fig. 1:**
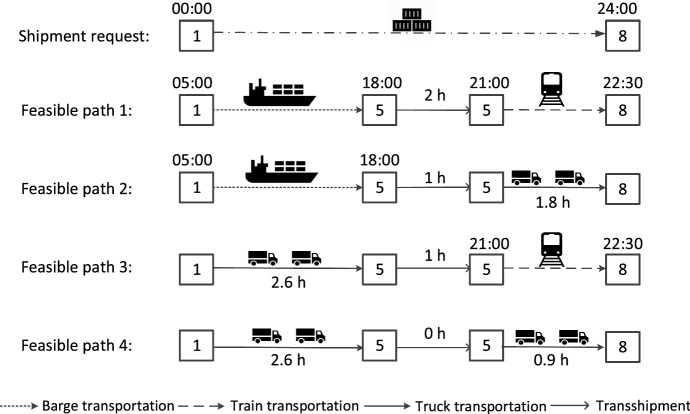
An illustrative example of shipment matching with different paths

An illustrative example of shipment matching with feasible paths is shown in Fig. 1. Here, a shipment needs to be transported from origin terminal 1 to destination terminal 8 after release time 00:00, and the due time of the shipment is 24:00. The shipment can be matched with different paths (i.e., service combinations). Using feasible path 1, the shipment will be loaded at origin terminal 1 and transported by a barge service to transshipment terminal 5, and then the shipment is transferred to a train service which delivers the shipment to its destination terminal.

The notation used in this paper is presented in Table 2.

**Table 2:** Notation

*Sets*	
*R*	Shipment requests received within a planning horizon, $R={\hat{R}}^0 \cup {\hat{R}}^1 \cup ...\cup {\hat{R}}^T$
${\hat{R}}^0$	Contractual requests that are received before the planning horizon
${\hat{R}}^t$	Spot requests that are received during time interval $\left( t-1,t\right]$, $t>0$
$R^{t}$	Shipment requests that are received before stage *t* and will expire before stage $t+1$
${\bar{R}}^t$	Shipment requests that are active at stage *t*
$\omega ^{rk}$	Set of sampled requests received at stage $k \in K=\{t+1,...,\min \{t+H,T\}\}$ under scenario
	$$\gamma \in \{1,...,\varGamma \}$$
*V*	Transport services within a planning horizon, $V= V^{\mathrm {barge}} \cup V^{\mathrm {train}} \cup V^{\mathrm {truck}}$
*P*	Feasible paths
$P_r$	Feasible paths for shipment *r*
$P_{rv}$	Feasible paths for shipment *r* including service *v*
*N*	Terminals
*Parameters*	
$o_{r}$	Origin terminal of shipment request $r \in R$
$d_{r}$	Destination terminal of shipment request $r \in R$
$u_{r}$	Container volume of shipment request $r \in R$
$t_{r}^{\mathrm {announce}}$	Announce time of shipment request $r \in R$
$t_{r}^{\mathrm {release}}$	Release time of shipment request $r \in R$
$t_{r}^{\mathrm {due}}$	Due time of shipment request $r \in R$
$t_{r}^{\mathrm {expire}}$	Expiry date of shipment request $r \in R$
$o_{v}$	Origin terminal of service $v \in V, o_v \in N$
$d_{v}$	Destination terminal of service $v\in V, d_v\in N$
$t^{\mathrm {depature}}_v$	Departure time of time-scheduled service $v \in V^{\mathrm {barge}} \cup V^{\mathrm {train}}$
$t^{\mathrm {arrival}}_v$	Arrival time of time-scheduled service $v \in V^{\mathrm {barge}} \cup V^{\mathrm {train}}$
$t_v^{\mathrm {truck}}(\tau )$	Time-dependent travel time of truck service $v \in V^{\mathrm {truck}}$
$c_{v}$	Transport cost of service $v \in V$ per container
$e_{v}$	Carbon emissions of service $v \in V$ per container
$U_{v}^t$	Free capacity of service $v \in V^{\mathrm {barge}} \cup V^{\mathrm {train}}$ at stage $t \in \{0,1,...,T\}$
$c_{rp}$	The cost of matching request $r \in R$ with path $p \in P$
*T*	The planning horizon, $t \in \{0,1,...,T\}$
$\varGamma$	Number of scenarios
*H*	Length of prediction horizon
$N^{\mathrm {iteration}}$	Maximum iteration number
${\bar{x}}_{rp}^t$	The ‘overall design vector’ for request $r\in R^{t}$ matching with path $p\in P$
${\bar{y}}_{rp}^t$	The ‘overall design vector’ for request $r\in {\bar{R}}^t \backslash R^{t}$ matching with path $p\in P$
$\lambda _{rp}^{t\gamma }$	Lagrangian multipliers for request $r\in R^{t}$ matching with path $p\in P$
${\tilde{\lambda }}_{rp}^{t\gamma }$	Lagrangian multipliers for request $r\in {\bar{R}}^t \backslash R^{t}$ matching with path $p\in P$
$\rho _{rp}^{t\gamma }$	Penalty factors for request $r\in R^{t}$ matching with path $p\in P$
${\tilde{\rho }}_{rp}^{t\gamma }$	Penalty factors for request $r\in {\bar{R}}^t \backslash R^{t}$ matching with path $p\in P$
$\eta$	A small positive number designed to control the termination of simulations
$\alpha$	A constant designed to control the updating rate of penalty factors
*Random variables*	
$R^{\mathrm {spot}}$	Spot requests received over the planning horizon. The probability distributions
	$\{\pi _o, \pi _d, \pi _u, \pi _{t^{\mathrm {announce}}}, \pi _{t^{\mathrm {release}}}, \pi _{t^{\mathrm {due}}}, \pi _{t^{\mathrm {expire}}}\}$ are assumed known
*Decision variables*	
$x_{rp}^t$	A binary variable equal to 1 if request $r \in R^{t}$ is matched with path $p \in P$, 0 otherwise
$y_{rp}^t$	A binary variable equal to 1 if request $r \in {\bar{R}}^t \backslash R^{t}$ is matched with path $p \in P$, 0 otherwise
$z_{rp}^{\gamma k}$	A binary variable equal to 1 if request $r \in \omega ^{\gamma k}$ is matched with path $p \in P$ under scenario
	$\gamma \in \{1,...,\varGamma \}$ at stage $k \in K$, 0 otherwise
$x_{rp}^{t\gamma }$	Binary variable; 1 if request $r \in R^t$ is matched with path $p \in P$ under scenario $\gamma$
$y_{rp}^{t\gamma }$	Binary variable; 1 if request $r \in {\bar{R}}^t \backslash R^t$ is matched with path $p \in P$ under scenario $\gamma$

## Solution approaches

**Fig. 2:**
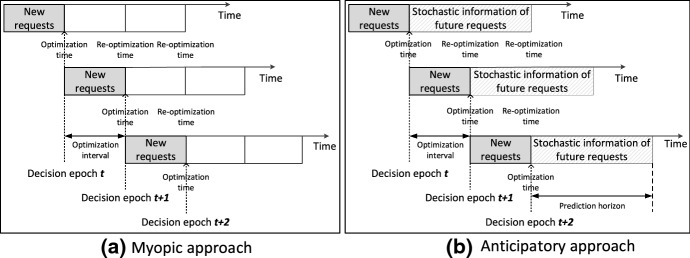
Illustration of the myopic approach and the anticipatory approach

In this section, we propose an anticipatory approach (AA) to solve the DSSM problem and use the myopic approach (MA) proposed by Guo et al. (2020) as a benchmark. Both the AA and the MA are implemented under a rolling horizon framework. However, the MA is based on deterministic information only while the AA incorporates stochastic information of future requests at each decision epoch, as shown in Fig. 2.

### Myopic approach

The MA presented in Guo et al. (2020) utilizes a rolling horizon framework to handle dynamic events, which is known as an efficient periodic re-optimization approach for dynamic problems (e.g., Arslan et al. 2019; Najmi et al. 2017; Wang and Kopfer 2015; Yang et al. 2004). The planning horizon is rolled forward to incorporate the dynamically released information, and the process continues until the end of the horizon. Under the MA, the system is optimized periodically at pre-specified points in time called *optimization times* (i.e., decision epochs). Let ${\hat{R}}^t=\{r\in R| t-1<t^{\mathrm {announce}}_r \le t\}$ be the set of spot requests received during time interval $\left( t-1,t\right]$, $t>0$. At decision epoch *t*, decisions for all active shipment requests ${\bar{R}}^t$ are made. Request *r* is active if it is already announced but not expired yet, formally ${\bar{R}}^t=\{r\in R| t_r^{\mathrm {announce}} \le t, t_r^{\mathrm {expire}} > t\}$. However, the decision for request $r \in {\bar{R}}^{t}$ is fixed only if $r \in R^t=\{r\in R| t_r^{\mathrm {announce}} \le t, t < t_r^{\mathrm {expire}} \le t+1\}$, namely the request will expire before the next decision epoch. The platform will inform shippers the decisions only if a match is fixed for them. Thus, only the matches fixed at stage *t* have effects on the free capacity of service $v \in V^{\mathrm {barge}} \cup V^{\mathrm {train}}$ at stage $t+1$.

We define $x_{rp}^t$ as the binary variable which is 1 if request in $r \in R^t$ is matched with path $p \in P$ and define $y_{rp}^t$ as the binary variable which is 1 if request in $r \in {\bar{R}}^t \backslash R^t$ is matched with path $p \in P$. Let $P_{rv}$ be the set of feasible paths for shipment request *r* including service *v*, $P_{rv}=\{p\in P_r| v\in p\}$. Under the MA, the objective function is to minimize the total costs of the current-stage decisions made for active requests ${\bar{R}}^t$. The formulation of the DSSM problem at stage $t \in \{0,1,...,T\}$ under the MA is:

1 $$\begin{aligned} \mathbf{P1} \ \ \ \min _{x^t,y^t}\sum _{r \in R^t} \sum _{p \in P_r} c_{rp}x_{rp}^t+\sum _{r \in {\bar{R}}^t \backslash R^t} \sum _{p \in P_r} c_{rp}y_{rp}^t \end{aligned}$$

subject to

2 $$\begin{aligned}&\sum _{p \in P_r} x_{rp}^t=1, \quad \forall r \in R^{t}, \end{aligned}$$

3 $$\begin{aligned}&\sum _{p \in P_r} y_{rp}^t=1, \quad \forall r \in {\bar{R}}^t \backslash R^t, \end{aligned}$$

4 $$\begin{aligned}&\sum _{r \in R^{t}}\sum _{p \in P_{rv}} u_rx_{rp}^t +\sum _{r \in {\bar{R}}^t \backslash R^t}\sum _{p \in P_{rv}} u_ry_{rp}^t \le U_v^t, \quad \forall v \in V^{\mathrm {barge}} \cup V^{\mathrm {train}}, \end{aligned}$$

5 $$\begin{aligned}&U_v^{t+1}=U_v^{t}-\sum _{r \in R^{t}}\sum _{p \in P_{rv}}u_rx_{rp}^{t}, \quad \forall v \in V^{\mathrm {barge}} \cup V^{\mathrm {train}}, \end{aligned}$$

6 $$\begin{aligned}&x_{rp}^t \in \{0,1\}, \quad \forall r\in R^{t}, p \in P, \end{aligned}$$

7 $$\begin{aligned}&y_{rp}^t \in \{0,1\}, \quad \forall r\in {\bar{R}}^t \backslash R^t, p \in P. \end{aligned}$$

Constraints (2–3) ensure that each request will be matched with one feasible path only. Constraints (4) ensure that the total container volumes of shipments assigned to service $v \in V^{\mathrm {barge}} \cup V^{\mathrm {train}}$ does not exceed its free capacity at stage *t*. Constraints (5) represent that the free capacity of service $v \in V^{\mathrm {barge}} \cup V^{\mathrm {train}}$ at the next stage is only influenced by the free capacity of service *v* at the current stage and the matching decisions made for requests $R^t$ which will expire before the next stage.

### Anticipatory approach

In this section, we propose the AA to incorporate the stochastic information of future requests at each decision epoch of the rolling horizon framework, in contrast to the MA in which dynamic decisions are made based on deterministic information only. The implementation of the AA for a synchromodal matching system is shown in Algorithm 1. Before the planning horizon, the system applies the preprocessing of feasible path to get the set of feasible paths. At each decision epoch of the rolling horizon framework, the system generates scenarios of future requests by randomly sampling from their probability distributions, applies the preprocessing procedure to obtain feasible matches for active requests and sampled requests, utilizes a sample average approximation method presented in Sect. 4.2.1 to get deterministic formulations, and utilizes a progressive hedging algorithm presented in Sect. 4.2.2 to generate solutions. The state of the system is updated based on the decisions made for requests $R^t$. Then the system is rolled forward to obtain the decisions for the next stage.

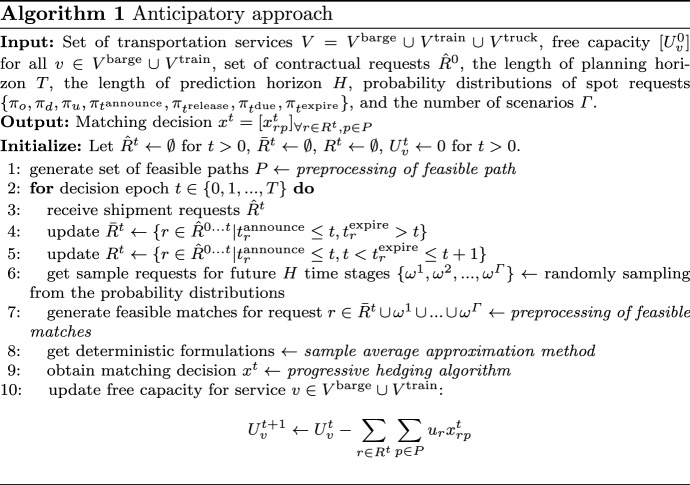



#### Sample average approximation method

The sample average approximation method is an approach to solve stochastic optimization problems by generating scenarios. In this technique, the expected objective function is approximated by a sample average estimate derived from a random sample (Verweij et al. 2003). At decision epoch *t*, a sample $\{\omega ^1,\omega ^2,...,\omega ^{\gamma },...,\omega ^{\varGamma }\}$ of $\varGamma$ scenarios is generated by randomly sampling from the probability distributions of spot requests $\{\pi _o,\pi _d,\pi _u,\pi _{t^{\mathrm {announce}}},\pi _{t^{\mathrm {release}}},\pi _{t^{\mathrm {due}}},\pi _{t^{\mathrm {expire}}}\}$. For companies that do not have accurate probability distributions, scenarios can also be sampled randomly from their historical operational data. Each scenario includes a realization of shipment requests from stage $t+1$ to stage $t+H$, $\omega ^{\gamma }=\{\omega ^{\gamma (t+1)},\omega ^{\gamma (t+2)},...,\omega ^{\gamma (t+H)}\}$. Here, *H* is the prediction horizon that is just long enough to obtain good decisions at stage *t*. The expected cost over the prediction horizon is approximated by the sample average function $\frac{1}{\varGamma }\sum _{\gamma =1}^{\varGamma }\sum _{k=t+1}^{t+H}\sum _{r \in \omega ^{\gamma k}}\sum _{p \in P_r} c_{rp}z_{rp}^{{\gamma }k}$, which is an unbiased estimator of future costs as the sample size $\varGamma$ goes to infinity and the prediction horizon $t+H=T$ (Ruszczyński and Shapiro 2003). We define *K* as the set of predicted time stages at stage *t*, $K=\{t+1,...,\min \{t+H,T\}\}, \forall t \in \{0,1,...,T-1\}$; $K=\emptyset \ \mathrm {when} \ t=T$. Let $z_{rp}^{\gamma k}$ be the binary variable which equals to 1 if request $r\in \omega ^{\gamma k}$ is matched with path $p \in P$ under scenario $\gamma \in \{1,..,\varGamma \}$ at stage $k \in K$. The formulation of the DSSM problem at stage *t* changes to:

8 $$\begin{aligned} \mathbf{P2} \ \min _{x^t,y^t,z^t}\sum _{r \in R^t} \sum _{p \in P_r} c_{rp}x_{rp}^{t}+\sum _{r \in {\bar{R}}^t \backslash R^t} \sum _{p \in P_r} c_{rp}y_{rp}^t+\frac{1}{\varGamma }\sum _{\gamma =1}^{\varGamma }\sum _{k \in K}\sum _{r \in \omega ^{\gamma k}}\sum _{p \in P_r} c_{rp}z_{rp}^{{\gamma }k} \end{aligned}$$

subject to Constraints (2–3, 5–7),

9 $$\begin{aligned}&\sum _{p \in P_r} z_{rp}^{{\gamma }k}=1, \quad \forall \gamma \in \{1,...,\varGamma \}, k\in K, r \in \omega ^{\gamma k}, \end{aligned}$$

10 $$\begin{aligned}&\sum _{r \in R^{t}}\sum _{p \in P_{rv}} u_rx_{rp}^t +\sum _{r \in {\bar{R}}^t \backslash R^t}\sum _{p \in P_{rv}} u_ry_{rp}^t+\sum _{k \in K}\sum _{r \in \omega ^{\gamma k}}\sum _{p \in P_{rv}} u_rz_{rp}^{{\gamma }k} \le U_v^t, \nonumber \\&\quad \forall \gamma \in \{1,...,\varGamma \}, v \in V^{\mathrm {barge}} \cup V^{\mathrm {train}}, \end{aligned}$$

11 $$\begin{aligned}&z_{rp}^{{\gamma }k} \in \{0,1\}, \forall \gamma \in \{1,...,\varGamma \}, k\in K, r \in \omega ^{\gamma k}, p \in P. \end{aligned}$$

In formulation $\mathbf{P2}$, $x^t$ and $y^t$ are first-stage decisions which do not depend on the scenarios, $z^{t}$ is the second-stage decision which depends on the corresponding scenarios. However, only $x^t$ will be implemented at each decision epoch, $y^t$ and $z^{t}$ will be released after the optimization.

#### Progressive hedging algorithm

Formulation $\mathbf{P2}$ is a large-scale deterministic binary integer program which is non-convex and highly complex to solve. In this section, we apply the progressive hedging algorithm (PHA) to solve the formulation. The PHA is first proposed by Rockafellar and Wets (1991) and has been implemented in many applications, such as stochastic network design problems (Crainic et al. 2014) and stochastic resource allocation problems (Watson and Woodruff 2010). It is a horizontal decomposition method which decomposes $\mathbf{P2}$ by scenarios rather than by time stages, and iteratively solves penalized version of the scenario-based subproblems to gradually enforce implementability (also called non-anticipativity) (Gade et al. 2016).

In $\mathbf{P2}$, the condition that the first-stage decisions $x^t$, $y^t$ must not depend on the realization of random variables is implicit. In the PHA scheme, we write the non-anticipativity constraints explicitly. We define $x_{rp}^{t\gamma }$ as the binary variable which equals to 1 if request $r \in R^t$ is matched with path $p \in P$ under scenario $\gamma$, $y_{rp}^{t\gamma }$ as the binary variable which equals to 1 if request $r \in {\bar{R}}^t \backslash R^t$ is matched with path $p \in P$ under scenario $\gamma$. Let ${\bar{x}}^{t}$ and ${\bar{y}}^t$ be the ‘overall design vector’. The DSSM problem is then reformulated as:

12 $$\begin{aligned} \mathbf{P3} \ \ \min _{x^{t},y^{t},z^t}\frac{1}{\varGamma }\sum _{\gamma =1}^{\varGamma }\left( \sum _{r \in R^t} \sum _{p \in P_r} c_{rp}x_{rp}^{t\gamma }+\sum _{r \in {\bar{R}}^t \backslash R^t} \sum _{p \in P_r} c_{rp}y_{rp}^{t\gamma }+\sum _{k \in K}\sum _{r \in \omega ^{\gamma k}}\sum _{p \in P_r} c_{rp}z_{rp}^{{\gamma }k}\right) \end{aligned}$$

subject to

13 $$\begin{aligned}&\sum _{p \in P_r} x_{rp}^{t\gamma }=1, \quad \forall \gamma \in \{1,...,\varGamma \}, r \in R^{t}, \end{aligned}$$

14 $$\begin{aligned}&\sum _{p \in P_r} y_{rp}^{t\gamma }=1, \quad \forall \gamma \in \{1,...,\varGamma \}, r \in {\bar{R}}^{t} \backslash R^t, \end{aligned}$$

15 $$\begin{aligned}&\sum _{p \in P_r} z_{rp}^{{\gamma }k}=1, \quad \forall \gamma \in \{1,...,\varGamma \}, k\in K, r \in \omega ^{\gamma k}, \end{aligned}$$

16 $$\begin{aligned}&\sum _{r \in R^{t}}\sum _{p \in P_{rv}} u_rx_{rp}^{t\gamma } +\sum _{r \in {\bar{R}}^t \backslash R^t}\sum _{p \in P_{rv}} u_ry_{rp}^{t\gamma }+\sum _{k \in K}\sum _{r \in \omega ^{\gamma k}}\sum _{p \in P_{rv}} u_rz_{rp}^{{\gamma }k} \le U_v^t, \nonumber \\&\quad \forall \gamma \in \{1,...,\varGamma \}, v \in V^{\mathrm {barge}} \cup V^{\mathrm {train}}, \end{aligned}$$

17 $$\begin{aligned}&x_{rp}^{t\gamma }={\bar{x}}_{rp}^{t}, \quad \forall \gamma \in \{1,...,\varGamma \}, r \in R^{t}, p\in P_r, \end{aligned}$$

18 $$\begin{aligned}&y_{rp}^{t\gamma }={\bar{y}}_{rp}^{t}, \quad \forall \gamma \in \{1,...,\varGamma \}, r \in {\bar{R}}^t \backslash R^t, p\in P_r, \end{aligned}$$

19 $$\begin{aligned}&U_v^{t+1}=U_v^{t}-\sum _{r \in R^{t}}\sum _{p \in P_{rv}}u_r{\bar{x}}_{rp}^{t}, \quad \forall v \in V^{\mathrm {barge}} \cup V^{\mathrm {train}}, \end{aligned}$$

20 $$\begin{aligned}&x_{rp}^{t\gamma } \in \{0,1\}, \quad \forall \gamma \in \{1,...,\varGamma \}, r\in R^{t}, p \in P, \end{aligned}$$

21 $$\begin{aligned}&y_{rp}^{t\gamma } \in \{0,1\}, \quad \forall \gamma \in \{1,...,\varGamma \}, r\in {\bar{R}}^t \backslash R^t, p \in P, \end{aligned}$$

22 $$\begin{aligned}&z_{rp}^{{\gamma }k} \in \{0,1\}, \forall \gamma \in \{1,...,\varGamma \}, k \in K, r \in \omega ^{\gamma k}, p \in P. \end{aligned}$$

Constraints (17–18) are the non-anticipatory constraints which stipulate that in all feasible solutions, the first-stage decisions are not allowed to depend on scenarios. Therefore, the newly added variables do not affect the optimal solution, and thus **P3** is equivalent to **P2**.

Following the PHA scheme, we drop off the constant coefficient $\varGamma ^{-1}$, and move the non-anticipativity constraints (17–18) into the objective function based on augmented Lagrangian strategy, which yields the objective function as follows:

23 $$\begin{aligned} \begin{aligned}&\mathbf{P4} \ \min _{x^{t},y^{t},z^{t}}\sum _{\gamma =1}^{\varGamma } \left( \sum _{r \in R^t} \sum _{p \in P_r} c_{rp}x_{rp}^{t\gamma }+\sum _{r \in {\bar{R}}^t \backslash R^t} \sum _{p \in P_r} c_{rp}y_{rp}^{t\gamma }+\sum _{k \in K}\sum _{r \in \omega ^{\gamma k}}\sum _{p \in P_r} c_{rp}z_{rp}^{{\gamma }k} \right. \\&\quad +\sum _{r\in R^t}\sum _{p \in P_r} \lambda _{rp}^{t\gamma }\left( x_{rp}^{t\gamma }-{\bar{x}}_{rp}^{t}\right) +\frac{1}{2}\sum _{r\in R^t}\sum _{p \in P_r}\rho _{rp}^{t\gamma }\left( x_{rp}^{t\gamma }-{\bar{x}}_{rp}^{t}\right) ^2\\&\quad \left. +\sum _{r \in {\bar{R}}^t \backslash R^t}\sum _{p \in P_r} {\tilde{\lambda }}_{rp}^{t\gamma }\left( y_{rp}^{t\gamma }-{\bar{y}}_{rp}^{t}\right) +\frac{1}{2}\sum _{r \in {\bar{R}}^t \backslash R^t}\sum _{p \in P_r} {\tilde{\rho }}_{rp}^{t\gamma }\left( y_{rp}^{t\gamma }-{\bar{y}}_{rp}^{t}\right) ^2\right) \end{aligned} \end{aligned}$$

subject to Constraints (13–16, 19–22).

In formulation **P4**, $\lambda _{rp}^{t\gamma }$ and ${\tilde{\lambda }}_{rp}^{t\gamma }$ are Lagrangian multipliers, $\rho _{rp}^{t\gamma }$ and ${\tilde{\rho }}_{rp}^{t\gamma }$ are penalty factors. Given the binary requirements for variables $x^t, y^t, {\bar{x}}^t, {\bar{y}}^t$, the objective function can be further formulated as:

24 $$\begin{aligned} \begin{aligned}&\mathbf{P5} \ \ \min _{x^{t},y^{t},z^{t}}\sum _{\gamma =1}^{\varGamma } \left( \sum _{r \in R^t} \sum _{p \in P_r} \left( c_{rp}+\lambda _{rp}^{t\gamma }+\frac{1}{2}\rho _{rp}^{t\gamma }-\rho _{rp}^{t\gamma }{\bar{x}}_{rp}^t\right) x_{rp}^{t\gamma }-\lambda _{rp}^{t\gamma }{\bar{x}}_{rp}^{t\gamma }+\frac{1}{2}\rho _{rp}^{t\gamma }{\bar{x}}_{rp}^{t}\right. \\&\quad +\sum _{r \in {\bar{R}}^t \backslash R^t} \sum _{p \in P_r} \left( c_{rp}+{\tilde{\lambda }}_{rp}^{t\gamma }+\frac{1}{2}{\tilde{\rho }}_{rp}^{t\gamma }-{\tilde{\rho }}_{rp}^{t\gamma }{\bar{y}}_{rp}^t\right) y_{rp}^{t\gamma }-{\tilde{\lambda }}_{rp}^{t\gamma }{\bar{y}}_{rp}^{t\gamma }+\frac{1}{2}{\tilde{\rho }}_{rp}^{t\gamma }{\bar{y}}_{rp}^{t}\\&\quad \left. +\sum _{k \in K}\sum _{r \in \omega ^{\gamma k}}\sum _{p \in P_r} c_{rp}z_{rp}^{{\gamma }k}\right) \end{aligned} \end{aligned}$$

subject to Constraints (13–16, 19–22).

For a given overall design ${\bar{x}}^t$, ${\bar{y}}^t$, the relaxed formulation **P5** is separable on a scenario basis. As it contains $\varGamma$ scenarios, it can be broken down into $\varGamma$ individual subproblems. An arbitrary subproblem indexed by $\gamma \in \{1,...,\varGamma \}$ by dropping constant terms has the following form:

25 $$\begin{aligned} \begin{aligned}&\mathbf{P6} \ \min _{x^{t\gamma },y^{t\gamma },z^{t\gamma }} \sum _{r \in R^t} \sum _{p \in P_r} \left( c_{rp}+\lambda _{rp}^{t\gamma }+\frac{1}{2}\rho _{rp}^{t\gamma }-\rho _{rp}^{t\gamma }{\bar{x}}_{rp}^t\right) x_{rp}^{t\gamma }\\&\quad +\sum _{r \in {\bar{R}}^t \backslash R^t} \sum _{p \in P_r} \left( c_{rp}+{\tilde{\lambda }}_{rp}^{t\gamma }+\frac{1}{2}{\tilde{\rho }}_{rp}^{t\gamma }-{\tilde{\rho }}_{rp}^{t\gamma }{\bar{y}}_{rp}^t\right) y_{rp}^{t\gamma }\\&\quad +\sum _{k \in K}\sum _{r \in \omega ^{\gamma k}}\sum _{p \in P_r} c_{rp}z_{rp}^{{\gamma }k} \end{aligned} \end{aligned}$$

subject to

26 $$\begin{aligned}&\sum _{p \in P_r} x_{rp}^{t\gamma }=1, \quad \forall r \in R^{t}, \end{aligned}$$

27 $$\begin{aligned}&\sum _{p \in P_r} y_{rp}^{t\gamma }=1, \quad \forall r \in {\bar{R}}^{t} \backslash R^t, \end{aligned}$$

28 $$\begin{aligned}&\sum _{p \in P_r} z_{rp}^{{\gamma }k}=1, \quad \forall k\in K, r \in \omega ^{\gamma k}, \end{aligned}$$

29 $$\begin{aligned}&\sum _{r \in R^{t}}\sum _{p \in P_{rv}} u_rx_{rp}^{t\gamma } +\sum _{r \in {\bar{R}}^t \backslash R^t}\sum _{p \in P_{rv}} u_ry_{rp}^{t\gamma }+\sum _{k \in K}\sum _{r \in \omega ^{\gamma k}}\sum _{p \in P_{rv}} u_rz_{rp}^{{\gamma }k} \le U_v^t, \nonumber \\&\quad \forall v \in V^{\mathrm {barge}} \cup V^{\mathrm {train}}, \end{aligned}$$

30 $$\begin{aligned}&x_{rp}^{t\gamma } \in \{0,1\}, \quad \forall r\in R^{t}, p \in P, \end{aligned}$$

31 $$\begin{aligned}&y_{rp}^{t\gamma } \in \{0,1\}, \quad \forall r\in {\bar{R}}^t \backslash R^t, p \in P, \end{aligned}$$

32 $$\begin{aligned}&z_{rp}^{{\gamma }k} \in \{0,1\}, \quad \forall k \in K, r\in \omega ^{\gamma k}, p \in P. \end{aligned}$$

Formulation $\mathbf{P6}$ is a scenario-based binary integer program which can be solved by using commercial solvers within an acceptable computational time, such as CPLEX. For a given scenario subproblem $\gamma$, the Lagrangian multiplier $\lambda _{rp}^{t\gamma }$ (${\tilde{\lambda }}_{rp}^{t\gamma }$) and the penalty parameter $\rho _{rp}^{t\gamma }$ (${\tilde{\rho }}_{rp}^{t\gamma }$) contribute to penalize the difference in terms of values between the local variable $x_{rp}^{t\gamma }$ ($y_{rp}^{t\gamma }$) and the current overall design ${\bar{x}}_{rp}^{t}$ (${\bar{y}}_{rp}^{t}$).

The pseudocode of the PHA at each decision epoch is shown in Algorithm 2. Each iteration of the PHA involves an optimization (Step 2) for scenario-based subproblems, an aggregation (Step 3) which corresponds to a projection of the individual scenario solutions onto the subspace of non-anticipative policies, a termination criteria (Step 4) to make sure the algorithm converges to within a tolerance, and a modification (Step 5) to update multipliers.

The key to success in implementing the PHA under a rolling horizon framework is to choose a proper $\rho$-value to avoid slow convergence. However, in the literature, there are no conclusive results on the selection of $\rho$-value. In this paper, we choose the $\rho$ in proportion to the matching cost of the associated request and path, namely $\rho ^t_{rp}=\alpha c_{rp}$ for $r\in R^t$, $p\in P$. This method will be evaluated in the experiments in comparison to a commonly used method in container transportation $\rho ^{n+1}=\alpha \rho ^n$ (Crainic et al. 2011; Dong et al. 2015).

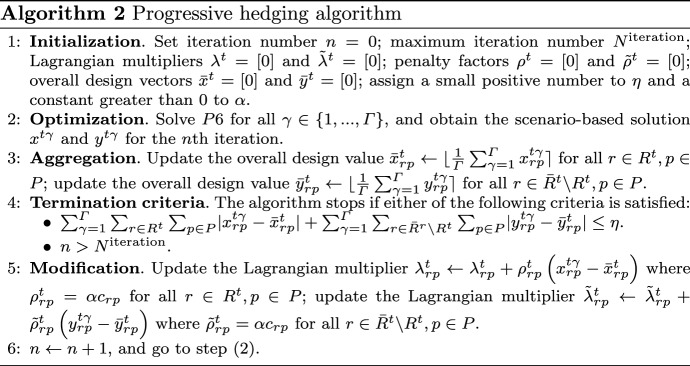



## Numerical experiments

In this section, we evaluate the performance of the anticipatory approach (AA) on the DSSM problem in comparison to the myopic approach (MA) proposed by Guo et al. (2020) and the commonly used greedy approach (GA) in the container transport industry (van Riessen et al. 2016). The GA is sometimes also referred to as a first come first served approach (Meng et al. 2019). Under the GA, a shipment request is assigned to the cheapest feasible path at the time of request arrival. To provide a theoretical lower bound of the AA, we also report the optimal solutions obtained when all the input information is known beforehand. The approaches are implemented in MATLAB, and all experiments are executed on 3.70 GHz Intel Xeon processors with 32 GB of RAM. The optimization problems are solved with CPLEX 12.6.3.

### Experimental setup

**Fig. 3:**
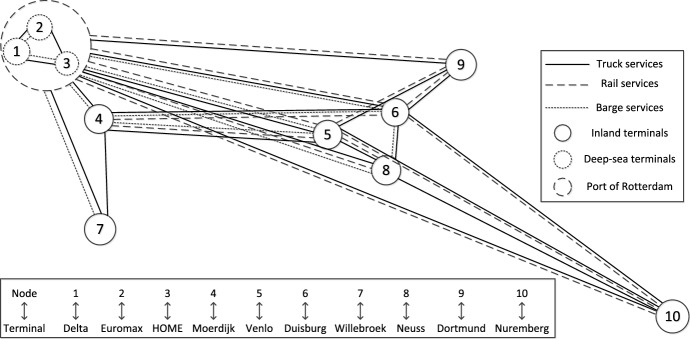
The topology of the hinterland synchromodal network derived from Guo et al. (2020)

In this paper, we use the hinterland synchromodal network designed by Guo et al. (2020) for the numerical experiments, which includes 3 deep-sea terminals in the port of Rotterdam (i.e., node 1, 2, and 3) and 7 inland terminals in the Netherlands, Belgium, and Germany (i.e., node 4, 5, 6, 7, 8, 9, and 10), as shown in Fig. 3. The network consists of 116 services, including 49 barge services, 33 train services, and 34 truck services. The detailed information of the services is presented in the Appendix.

We generate several instances to represent different characteristics of shipment requests within a given planning horizon. Each shipment request is characterized by its origin, destination, container volume, announce time, release time, expiry date, and due time. We assume that:

- the origins of shipments are independent and identically distributed among $\{1,2,3\}$ with probabilities $\{0.66,0.2,0.14\}$; the destinations of shipments are independent and identically distributed among $\{4,5,6,7,8,9,10\}$ with probabilities $\{0.306,0.317,0.153,0.076,0.071,0.034,0.043\}$;
- the container volumes of shipment requests which arrive before the planning horizon (i.e., contractual requests) are drawn independently from a uniform distribution with range [10, 30], the average container volume of contractual requests $U_1^{\mathrm {AVE}}=20$; the container volumes of spot requests are drawn independently from uniform distributions with range [1, 9], the average container volume of spot requests $U_2^{\mathrm {AVE}}=5$;
- the announce time of contractual requests is 0, while the frequency of spot requests arriving in the system belongs to Poisson distributions with mean $AT^{\mathrm {AVE}}$;
- the release time of contractual requests is drawn independently from a uniform distribution with range [1, 120]; the release time of spot requests is generated based on its announce time, $t_{r}^{\mathrm {release}}=\lceil t_{r}^{\mathrm {announce}} \rceil +\varDelta T$, $\varDelta T$ belongs to a uniform distribution with range [1, 6]; the expiry date is equal to the release time;
- the due time of shipment requests is generated based on its release time and lead time, $t_{r}^{\mathrm {due}}=t_{r}^{\mathrm {release}}+LD_r$, the lead time of shipments is independent and identically distributed among $\{24,48,72\}$ (unit: hours) with probabilities $\{0.15,0.6,0.25\}$. The delay cost coefficients of shipments with lead time 24, 48, and 72 h are 100, 70, and 50 €/h-TEU, respectively.

**Fig. 4:**
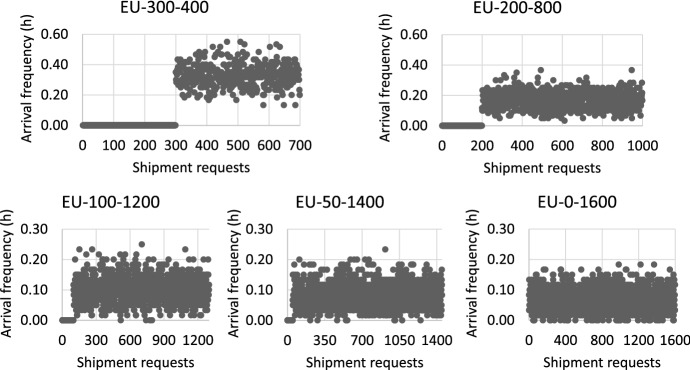
Arrival frequency of instances

We use $\mathrm {EU}-n_1-n_2$ to represent an instance with $n_1$ contractual requests and $n_2$ spot requests. We set $AT^{\mathrm {AVE}}$ to 20, 10, 6, 5, and 4 min (i.e., about 0.33, 0.17, 0.1, 0.08, and 0.07 h per request) for instances EU-300-400, EU-200-800, EU-100-1200, EU-50-1400, and EU-0-1600, respectively, as shown in Fig. 4. We define the degree of dynamism as the ratio between the number of containers from spot requests and the total number of containers, namely, degree of dynamism=$\frac{n_2*U_2^{\mathrm {AVE}}}{n1*U_1^{\mathrm {AVE}}+n2*U_2^{\mathrm {AVE}}}$. Therefore, the degrees of dynamism for instances EU-300-400, EU-200-800, EU-100-1200, EU-50-1400, and EU-0-1600 are 25%, 50%, 75%, 87.5%, and 100%, respectively.

The length of the planning horizon is set to 168 h for all the instances. The length of the optimization interval is set to 1 h in the MA and the AA. At each decision epoch of the AA, a sample is generated randomly based on the probability distributions presented above. In case of sample instability, for each instance, we replicate the optimization process 10 times under the AA.

### Impact of the degree of dynamism

**Fig. 5:**
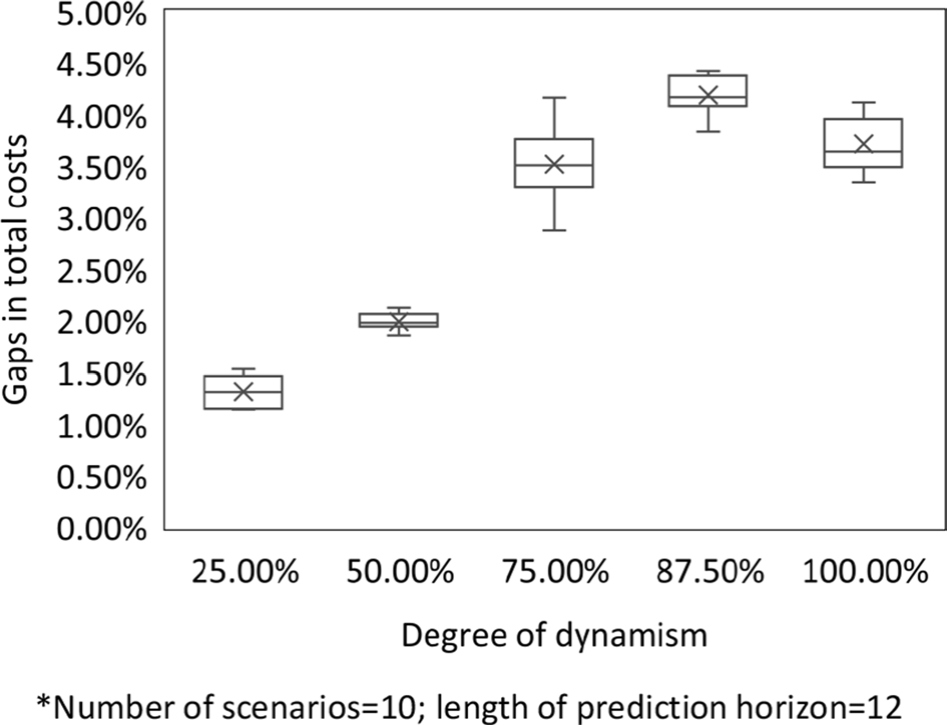
Impact of the degree of dynamism

To test the influence of the degree of dynamism, we set the number of scenarios to 10, and the length of prediction horizon to 12 h. We use ‘gaps in total costs’ as the performance indicator which is given by (benchmark value - objective value)/benchmark value. Here, the total cost generated by the MA is the benchmark value, while the total cost generated by the AA is the objective value. Therefore, the higher the ‘gaps in total costs’, the better the performance of the AA in reducing total costs. Fig. 5 shows that the AA has better performance than the MA in all the instances in reducing total costs, and the gap between the AA and the MA grows with the increasing of the degree of dynamism from 25% to 87.5%. Nevertheless, further increasing the degree of dynamism to 100%, the gap in total costs stays around 4%.

### Impact of the number of scenarios and the length of prediction horizon

**Fig. 6:**
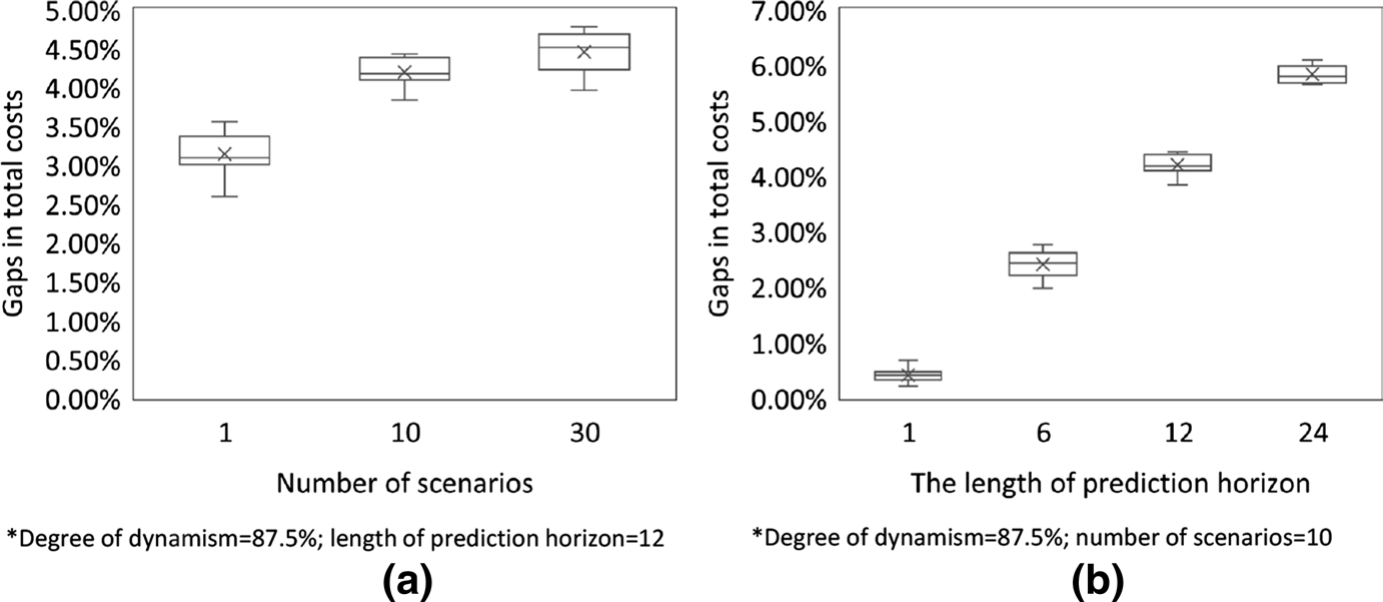
Impact of the number of scenarios and the length of prediction horizon

With regards to the number of scenarios, we set the degree of dynamism to 87.5% (i.e., instance EU-50-1400), and the length of prediction horizon to 12 h. The number of scenarios is varied from 1 to 30. Figure 6a shows that increasing the number of scenarios, the gap in total costs between the AA and the MA becomes larger. The reason is that the larger the number of scenarios, the more accurate the representation of the future. On the other hand, we set the number of scenarios to 10, and vary the length of prediction horizon from 1 to 24 h for instance EU-50-1400. Figure 6b shows that the length of prediction horizon has high influences on the performance of the AA in reducing total costs. The longer the prediction horizon, the more the stochastic information of future requests will be considered. The system thus reserves capacities for predicted future requests which are more ‘valuable’. In turn, the performance of the system over the planning horizon becomes better.

### Impact of the selection of $\rho$-value

To test the impact of the selection of $\rho$-value, we design 10 instances with different number of requests, different number of scenarios and different length of prediction horizon. The proposed cost proportional method (i.e., $\rho _{rp}=\alpha c_{rp}$) is evaluated in comparison to the typical iterative method (i.e., $\rho ^{n+1}={\hat{\alpha }} \rho ^n$). We set $\alpha =1, {\hat{\alpha }}=1.1, \rho ^0=1$. Table 3 shows that the costs generated by these two methods are almost the same in all the instances. However, the number of iterations (i.e., N. Iteration) and the computation time (i.e., CPU) under the typical iterative method are way much higher than the cost proportional method. The larger the number of scenarios and the length of prediction horizon, the higher the gaps between these two methods. We also notice that the CPU increases dramatically with the increase of shipment requests under the typical iterative method. In comparison, all these instances can be solved by the cost proportional method within 20 s. With the cost proportional method, the PHA can be implemented under a rolling horizon framework to provide timely solutions at each decision epoch.

**Table 3:** Impact of the selection of $\rho$-value

Instances	$\varGamma$	*H*	$\rho _{rp}=\alpha c_{rp}$			$\rho ^{n+1}={\hat{\alpha }} \rho ^n$		
			Costs (€)	N. iteration	CPU (s)	Costs (€)	N. iteration	CPU (s)
EU-50-0	5	6	144553	2	1.25	144549	35	29.43
EU-50-0	10	12	195831	3	2.66	195810	51	44.39
EU-50-0	10	24	283651	2	4.10	283654	50	99.60
EU-50-0	10	48	434789	3	17.95	434763	50	275.49
EU-50-0	30	12	189193	2	1.84	189194	48	137.82
EU-50-0	30	24	286633	2	4.10	286631	52	111.17
EU-50-0	30	48	438920	3	17.39	442021	94	589.13
EU-100-0	10	12	292268	2	2.20	292274	27	31.36
EU-200-0	10	12	422272	3	3.65	422273	69	87.17
EU-300-0	10	12	634021	5	13.06	632923	60	182.77

### Comparison between the GA, the MA, and the AA

In this section, we test the performance of the AA in comparison to the MA and the GA. While the result obtained from the GA provides an upper bound of the AA, we use the solutions obtained when all the input information is known in advance as the theoretical lower bounds. Specifically, we assume all the contractual and spot requests are received before the planning horizon, which gives rise to an optimization problem that includes all the shipments and services. Due to the computational complexity, the problem is solved by the heuristic algorithm designed in Guo et al. (2020). We set $\varGamma =100, H=48, N^{\mathrm {iteration}}=100, \alpha =1, \eta =0.001$ for the AA. The comparison between the GA, the MA, and the AA is shown in Table 4. We consider three performance indicators: the total costs (€), the ave. CPU (s), and the improvements. The ave. CPU of the GA, the MA, and the AA is the average computation time per stage over the planning horizon (i.e., 168 time stages). Although the AA needs to solve a large number of subproblems at each decision epoch due to the iteration of Lagrangian multipliers, applying the parallel computing techniques enables to use multiple CPUs to solve the subproblems in a single iteration of the AA simultaneously. We use the results obtained from the GA as the benchmark, the improvements between the MA/AA and the GA are given by (benchmark value - objective value)/benchmark value. Table 4 shows that the AA outperforms the GA and the MA in all the instances. While the MA has average improvements of about 2.37% in comparison to the GA, the AA has average improvements of about 6.12%. Impressively, we notice that with the designed AA, the gap between the AA and the theoretical lower bounds is no more than 2.65% on average.

**Table 4:** Comparison between the GA, the MA, and the AA

Instances	GA		MA			AA			Theoretical lower bounds		
	Costs	CPU	Costs	CPU	Improv (%)	Costs	CPU	Improv (%)	Costs	CPU	Improv (%)
EU-300-400	975784	0.02	965934	0.76	1.01	945127	64.42	3.14	937292	8.22	3.94
EU-200-800	971170	0.02	933842	0.59	3.84	912194	13.70	6.07	900644	16.98	7.26
EU-100-1200	1004494	0.02	980743	0.42	2.36	922322	29.49	8.18	914585	29.93	8.95
EU-50-1400	994406	0.02	972295	0.41	2.22	924220	44.12	7.06	892168	42.33	10.28
EU-0-1600	971799	0.01	948457	0.32	2.40	912294	62.04	6.12	841668	56.16	13.39
Average					2.37			**6.12**			8.77

## Conclusions and future research

In this paper, we introduced a dynamic and stochastic shipment matching (DSSM) problem in hinterland synchromodal transportation. The problem is considered dynamic since spot requests arrive in the system in real-time. The problem is considered stochastic since the information of spot requests is not known with certainty. To solve the problem, we developed an anticipatory approach (AA) which uses a sample average approximation method to address spot request uncertainties and a progressive hedging algorithm to generate solutions at each decision epoch of a rolling horizon framework.

We validated the performance of the AA on the DSSM problem in comparison with the myopic approach (MA) proposed by Guo et al. (2020) in which dynamic decisions are made based on deterministic information only and the greedy approach (GA) which is commonly used in practice. The experimental results indicate that the AA outperforms the GA and the MA in all the instances of the synchromodal matching system. Compared with the GA, the AA has total cost savings up to 8.18%.

From a managerial viewpoint, with the proposed AA, the utilization of barges, trains, and trucks can be managed more efficiently by taking into account the time-sensitivity of current received requests and the predicted future requests. Besides, the proposed approach enables the decision makers to dynamically update the decisions of the previously received shipments when the newly received ones can be better served with the previously matched services. This increases the adaptive nature of transport systems to meet today’s environment. Furthermore, the experimental results show that the more the stochastic information is incorporated, the better the performance of the AA. However, the computational complexity increases with the increase of stochastic information. To implement such an approach in practice, the trade-off between solution quality and computational complexity must be considered.

Future research can be conducted under three directions. First, due to the capacity limitation of road infrastructures, the number of trucks is limited in a synchromodal network. Therefore, the rejection of shipment requests can be considered in the online matching processes to avoid infeasible solutions. Another research direction is to investigate the benefits of incorporating ad hoc services (i.e., dynamic services). Considering the excess capacity of services from carriers, the online matching of contractual requests, spot requests, dedicated services, and ad hoc services gives rise to a new variant of the dynamic shipment matching problem in synchromodal transportation. Third, due to the existence of traffic congestion and terminal congestion in synchromodal transportation, travel time of services and transfer time at terminals are usually uncertain. Combining multiple uncertainties in dynamic shipment matching is a promising research direction.

## Appendix

The detailed information of barge, train and truck services is presented in Tables 5, 6 and 7. The barge and train connections are derived from European Gateway Services (http://www.europeangatewayservices.com/en/). We assume there exists truck connections between all the terminals. The distance of services used in this paper is obtained from European Gateway Services, InlandLinks (https://www.inlandlinks.eu/en), and Google maps.

**Table 5:** Barge services in the numerical experiments

Barge services	Origin	Destination	Capacity (TEU)	Departure time	Arrival time	Transit time (h)	Transit cost (€/TEU)	Distance (km)	Carbon emissions (kg/TEU)
1	Delta	Euromax	160	53	54	1	2.10	15	3.43
2	Delta	HOME	160	53	55.5	2.5	5.25	37.5	8.58
3	Delta	Moerdijk	160	3	8	5	10.50	75	17.16
4	Delta	Moerdijk	160	15	20	5	10.50	75	17.16
5	Delta	Moerdijk	160	27	32	5	10.50	75	17.16
6	Delta	Moerdijk	160	39	44	5	10.50	75	17.16
7	Delta	Moerdijk	160	51	56	5	10.50	75	17.16
8	Delta	Moerdijk	160	63	68	5	10.50	75	17.16
9	Delta	Moerdijk	160	75	80	5	10.50	75	17.16
10	Delta	Moerdijk	160	87	92	5	10.50	75	17.16
11	Delta	Moerdijk	160	99	104	5	10.50	75	17.16
12	Delta	Moerdijk	160	111	116	5	10.50	75	17.16
13	Delta	Moerdijk	160	123	128	5	10.50	75	17.16
14	Delta	Moerdijk	160	135	140	5	10.50	75	17.16
15	Delta	Moerdijk	160	147	152	5	10.50	75	17.16
16	Delta	Moerdijk	160	159	164	5	10.50	75	17.16
17	Delta	Venlo	160	12	25	13	27.30	195	44.62
18	Delta	Venlo	160	18	31	13	27.30	195	44.62
19	Delta	Venlo	160	36	49	13	27.30	195	44.62
20	Delta	Venlo	160	42	55	13	27.30	195	44.62
21	Delta	Venlo	160	60	73	13	27.30	195	44.62
22	Delta	Venlo	160	66	79	13	27.30	195	44.62
23	Delta	Venlo	160	90	103	13	27.30	195	44.62
24	Delta	Venlo	160	96	109	13	27.30	195	44.62
25	Delta	Venlo	160	120	133	13	27.30	195	44.62
26	Delta	Duisburg	160	82	98	16	33.60	240	54.91
27	Delta	Duisburg	160	102	118	16	33.60	240	54.91
28	Delta	Willebroek	160	68	79	11	23.10	165	37.75
29	Delta	Willebroek	160	98	109	11	23.10	165	37.75
30	Delta	Willebroek	160	146	157	11	23.10	165	37.75
31	Delta	Neuss	160	80	97	17	35.70	255	58.34
32	Euromax	Moerdijk	160	3	8.5	5.5	11.55	82.5	18.88
33	Euromax	Moerdijk	160	51	56.5	5.5	11.55	82.5	18.88
34	Euromax	Moerdijk	160	99	104.5	5.5	11.55	82.5	18.88
35	Euromax	Venlo	160	27	40.5	13.5	28.35	202.5	46.33
36	Euromax	Venlo	160	75	88.5	13.5	28.35	202.5	46.33
37	Euromax	Duisburg	160	103	119.5	16.5	34.65	247.5	56.63
38	Euromax	Willebroek	160	112	123.5	11.5	24.15	172.5	39.47
39	Euromax	Neuss	160	66	83.5	17.5	36.75	262.5	60.06
40	HOME	Moerdijk	160	5	8	3	6.30	45	10.30
41	HOME	Moerdijk	160	53	56	3	6.30	45	10.30
42	HOME	Moerdijk	160	101	104	3	6.30	45	10.30
43	HOME	Venlo	160	99	110	11	23.10	165	37.75
44	HOME	Venlo	160	126	137	11	23.10	165	37.75
45	HOME	Duisburg	160	51	66.5	15.5	32.55	232.5	53.20
46	HOME	Willebroek	160	20	30.5	10.5	22.05	157.5	36.04
47	Moerdijk	Venlo	160	95	105	10	21.00	150	34.32
48	Moerdijk	Duisburg	160	71	83	12	25.20	180	41.18
49	Duisburg	Neuss	160	120	122.5	2.5	5.25	37.5	8.58

**Table 6:** Train services in the numerical experiments

Train services	Origin	Destination	Capacity (TEU)	Departure time	Arrival time	Transit time (h)	Transit cost (€/TEU)	Distance (km)	Carbon emissions (kg/TEU)
1	Delta	Venlo	90	16	20	4	30.33	180	56.63
2	Delta	Venlo	90	40	44	4	30.33	180	56.63
3	Delta	Venlo	90	9	13	4	30.33	180	56.63
4	Delta	Venlo	90	33	37	4	30.33	180	56.63
5	Delta	Venlo	90	57	61	4	30.33	180	56.63
6	Delta	Venlo	90	81	85	4	30.33	180	56.63
7	Delta	Venlo	90	105	109	4	30.33	180	56.63
8	Delta	Venlo	90	129	133	4	30.33	180	56.63
9	Delta	Duisburg	90	41	47	6	44.73	270	84.94
10	Delta	Duisburg	90	75	81	6	44.73	270	84.94
11	Delta	Duisburg	90	99	105	6	44.73	270	84.94
12	Delta	Duisburg	90	113	119	6	44.73	270	84.94
13	Delta	Neuss	90	110	115	5	37.53	225	70.79
14	Delta	Dortmund	90	88	95	7	51.93	315	99.10
15	Delta	Nuremberg	90	51	66	15	109.53	675	212.36
16	Delta	Nuremberg	90	99	114	15	109.53	675	212.36
17	Euromax	Venlo	90	78	82.5	4.5	33.93	202.5	63.71
18	Euromax	Venlo	90	102	106.5	4.5	33.93	202.5	63.71
19	Euromax	Duisburg	90	75	81.5	6.5	48.33	292.5	92.02
20	Euromax	Duisburg	90	99	105.5	6.5	48.33	292.5	92.02
21	Euromax	Neuss	90	77	82.5	5.5	41.13	247.5	77.86
22	Euromax	Dortmund	90	78	85.5	7.5	55.53	337.5	106.18
23	Euromax	Nuremberg	90	79	94.5	15.5	113.13	697.5	219.43
24	HOME	Venlo	90	86	89.5	3.5	26.73	157.5	49.55
25	HOME	Duisburg	90	27	32.5	5.5	41.13	247.5	77.86
26	HOME	Duisburg	90	75	80.5	5.5	41.13	247.5	77.86
27	Moerdijk	Venlo	90	75	78	3	23.13	135	42.47
28	Moerdijk	Duisburg	90	77	81	4	30.33	180	56.63
29	Venlo	Neuss	90	112	113.5	1.5	12.33	67.5	21.24
30	Venlo	Dortmund	90	113	115.5	2.5	19.53	112.5	35.39
31	Venlo	Nuremberg	90	114	125	11	80.73	495	155.73
32	Duisburg	Dortmund	90	121	122.5	1.5	12.33	67.5	21.24
33	Duisburg	Nuremberg	90	122	132	10	73.53	450	141.57

**Table 7:** Truck services in the numerical experiments

Truck services	Origin	Destination	Transit time (h)	Transit cost (€/TEU)	Distance (km)	Carbon emissions (kg/TEU)
1	Delta	Euromax	0.2	92.00	15	13.30
2	Delta	HOME	0.5	115.40	37.5	33.25
3	Delta	Moerdijk	1.0	154.40	75	66.50
4	Delta	Venlo	2.6	279.20	195	172.89
5	Delta	Duisburg	3.2	326.00	240	212.78
6	Delta	Willebroek	2.0	232.40	150	132.99
7	Delta	Neuss	3.5	349.40	262.5	232.73
8	Delta	Dortmund	4.0	388.40	300	265.98
9	Delta	Nuremberg	9.0	778.40	675	598.46
10	Euromax	HOME	0.6	123.20	45	39.90
11	Euromax	Moerdijk	1.2	170.00	90	79.79
12	Euromax	Venlo	2.8	294.80	210	186.19
13	Euromax	Duisburg	3.3	333.80	247.5	219.43
14	Euromax	Willebroek	2.2	248.00	165	146.29
15	Euromax	Neuss	3.6	357.20	270	239.38
16	Euromax	Dortmund	4.2	404.00	315	279.28
17	Euromax	Nuremberg	9.5	817.40	712.5	631.70
18	HOME	Moerdijk	0.6	123.20	45	39.90
19	HOME	Venlo	2.3	255.80	172.5	152.94
20	HOME	Duisburg	2.7	287.00	202.5	179.54
21	HOME	Willebroek	1.5	193.40	112.5	99.74
22	HOME	Neuss	3.0	310.40	225	199.49
23	HOME	Dortmund	3.4	341.60	255	226.08
24	HOME	Nuremberg	8.8	762.80	660	585.16
25	Moerdijk	Venlo	1.8	216.80	135	119.69
26	Moerdijk	Duisburg	2.4	263.60	180	159.59
27	Moerdijk	Willebroek	1.4	175.20	95	84.23
28	Venlo	Duisburg	0.8	138.80	60	53.20
29	Venlo	Neuss	0.9	146.60	67.5	59.85
30	Venlo	Dortmund	1.5	193.40	112.5	99.74
31	Venlo	Nuremberg	6.6	591.20	495	438.87
32	Duisburg	Neuss	0.5	115.40	37.5	33.25
33	Duisburg	Dortmund	0.9	146.60	67.5	59.85
34	Duisburg	Nuremberg	6	544.40	450	398.97
